# EBF1 Regulates Cardiac Development Through Fibroblast to Myocyte Signaling

**DOI:** 10.3390/jcdd13090451

**Published:** 2026-09-10

**Authors:** Eugene E. Kim, Michael Gildea, Alireza Khodadadi-Jamayran, Fang-Yu Liu, Jie Zhang, Glenn I. Fishman

**Affiliations:** Leon H. Charney Division of Cardiology, NYU Grossman School of Medicine, New York, NY 10012, USA

**Keywords:** heart, development, EBF1, transcription factor, collagen, integrin, hyperplasia, PDGFRα

## Abstract

The transcription factor early B-cell factor 1 (EBF1) plays critical developmental roles in numerous organ systems, including B-cells, kidney, bone, and heart. During cardiogenesis, cardiomyocyte expression of EBF1 is reportedly undetectable and its effects on myocyte development and proliferation are thought to reflect a non-cell autonomous mechanism, acting via intercellular communication from EBF1 expressing non-myocyte cells. Here, using single-cell transcriptional profiling, we confirm the absence of Ebf1 transcripts in cardiomyocytes. Furthermore, employing computational receptor–ligand interaction analysis of dissociated cells from wildtype and EBF1-deficient hearts, we show that loss of function of this pioneer transcription factor enhances fibroblast to myocyte signaling via the collagen-integrin pathway. Finally, we generated fibroblast-specific EBF1 knockout mice using a PDGFRα-Cre transgenic driver, and found a nearly identical phenotype to that of the generalized knockout, with runting, premature death, an increase in left ventricular relative wall thickness and cardiomyocyte hyperplasia. These findings provide further mechanistic insight into the non-cell autonomous mechanism of action of EBF1 in cardiac growth and development.

## 1. Introduction

EBF1 and its orthologs, collectively the COE (Collier/Olf/EBF) family of transcription factors, represent an evolutionarily ancient lineage that plays a key role in cell lineage specification [1,2,3,4,5]. Acting as a pioneer factor [6,7], EBF1 regulates chromatin accessibility through interactions with the SWI/SNF chromatin remodeling complex [8] and directs the expression of genes crucial for the development of a variety of cell types. Since its original description in regulating B-cell differentiation [1], EBF1 expression has been detected in nearly all tissues, including heart [4,9], lung [9], kidney [2,10], liver [10], gastrointestinal tissue [11], adipose tissue [12], vasculature [13], skin [3,14], and bone [3]. Recent work into the role of EBF1 in cardiac development suggests a non-cell autonomous mechanism affecting cardiomyocyte proliferation and differentiation [4]. EBF1 expression was undetectable in cardiomyocytes, while robust expression was seen in the non-myocyte populations. Coculture experiments supported intercellular signaling from non-myocytes to myocytes as the basis for EBF1’s effects on myocyte development. Thus, analogous to the effects of EBF1 on bone development, where a non-cell autonomous mechanism is also implicated in regulating the behavior of osteoblasts [3], EBF1 is highly expressed in non-myocytes of the heart and regulates cardiomyocyte differentiation through intercellular signaling [4]. The current report delves further into the mechanistic basis through which EBF1 regulates heart development.

## 2. Materials and Methods

Mutant mice: Ebf1 knockout [1], PDGFRα-Cre (JAX stock #013148), floxed EBF1 [15], and PDGFRα-GFP [16] mice have previously been described. Mouse lines were maintained in a mixed genetic background.

Single-cell sequencing: Hearts were excised from embryonic day 16.5 animals euthanized via decapitation. Cells were dissociated as previously described [4]. Raw 10x Genomics single-cell RNA-seq data were processed with Cell Ranger v7.0.0 using the mouse reference genome GRCm38 with GENCODE vM23/Ensembl98 annotations. Filtered feature-barcode matrices were analyzed in R (v4.1.2) using Seurat (v4.4.0). High-quality cells were retained if they expressed >500 genes and had <25% mitochondrial transcripts. Normalization and variance stabilization were performed with SCTransform v2 [17]. To align similar cell states across conditions, Seurat’s anchor-based integration was used [18]. Principal component analysis was performed on the integrated data, and the top 30 PCs were used to construct a shared nearest neighbor graph for Louvain clustering with a resolution of 0.75. Marker genes of each cluster were identified using the Seurat function FindMarkers on SCTransform-normalized data. Differential expression between conditions was performed on SCTransform-normalized data using FindMarkers. Differentially expressed genes were defined using a Benjamini–Hochberg adjusted *p*-value (FDR) < 0.1 together with a >2-fold-change threshold. Normality was not assumed. Cell–cell communication analysis was performed using the CellChat R package (v2.1.2) [19].

Histology and Immunohistochemistry: Hearts were excised from postnatal day 17 animals euthanized via cervical dislocation or decapitation after isofluorane anesthesia. Hearts were fixed overnight in 4% paraformaldehyde and cryosections or paraffin-embedded sections were prepared and stained as previously described [20]. Primary antibodies were directed against β-catenin (Abcam) and visualized by confocal (Leica) microscopy. For cell size analysis, conditional knockout mice were first screened by echocardiography and those with an increase in relative wall thickness were chosen for analysis. For analysis of cell size, sections were stained with wheat germ agglutinin-conjugated Alexa 488 (Invitrogen) and cell size was quantified using ImageJ 1.54p. Investigators were blinded to animal genotypes for cell size assessment. Two-way ANOVA was performed for statistical analysis.

Echocardiograms: Echocardiograms were performed as previously described [21] (Vevo; FujiFilm VisualSonics Inc., Toronto, ON, Canada). Mice were anesthetized with inhaled 2% isoflurane. Heart rate was monitored, and core body temperature was maintained at 37.5 °C. Animals with a heart rate between 400 and 600 bpm were included for analysis. Echo measurements were performed in a blinded fashion and performed on a single representative systolic and diastolic frame. Unpaired Welch’s *t*-tests were used to establish *p*-values.

Bulk gene transcriptional profiling: Postnatal day 14 hearts were dissociated using Langendorf perfusion and FACS (Beckman Coulter MoFlo) was performed as previously described [21]. Total RNAs were extracted from GFP+ FACS sorted cells using the RNeasy or RNeasy Mini kit (Qiagen, Germantown, MD, USA). Sequencing libraries were prepared using the TruSeq RNA Library Prep Kit v2 (Illumina, San Diego, CA, USA). Samples were sequenced 50 bp paired-ended at 10 million to 20 million reads per replicate on an Illumina HiSeq. 2500 instrument. Library preparation and sequencing were performed at the New York University School of Medicine Genome Technology Center. All the reads were mapped to the mouse reference genome (mm10) using the STAR aligner (v2.5.0c) [22]. Alignments were guided by a Gene Transfer File (GTF, version GRCm38.74) and the mean read insert sizes, and their standard deviations were calculated using Picard tools (v.1.126) (http://broadinstitute.github.io/picard/; accessed on 18 February 2026). The read count tables were generated using HTSeq (v0.6.0) [23] and normalized based on their library size factors using DESeq. 2 (v3.0) [24], and differential expression analysis was performed. The Read Per Million (RPM) normalized BigWig files were generated using BEDTools (v2.17.0) [25] and bedGraphToBigWig tool (v4), and downstream statistical analyses and generating plots were performed in R environment (v3.1.1) (http://www.r-project.org/).

Gene set enrichment analysis (GSEA): Ranked list file format (rnk) files were made using log2 fold changes in all the genes and GSEA was performed using GSEA tool (https://www.gsea-msigdb.org/gsea/index.jsp) for the following gene sets (gmt file): c2.cp.kegg.v7.0.symbols.gmt (KEGG pathways), c5.mf.v7.0.symbols.gmt (molecular function), c5.bp.v7.0.symbols.gmt (biological process), h.all.v7.0.symbols.gmt (immunological signature), and c7.all.v7.0.symbols.gmt (cancer hallmark).

## 3. Results

**Figure 1:**
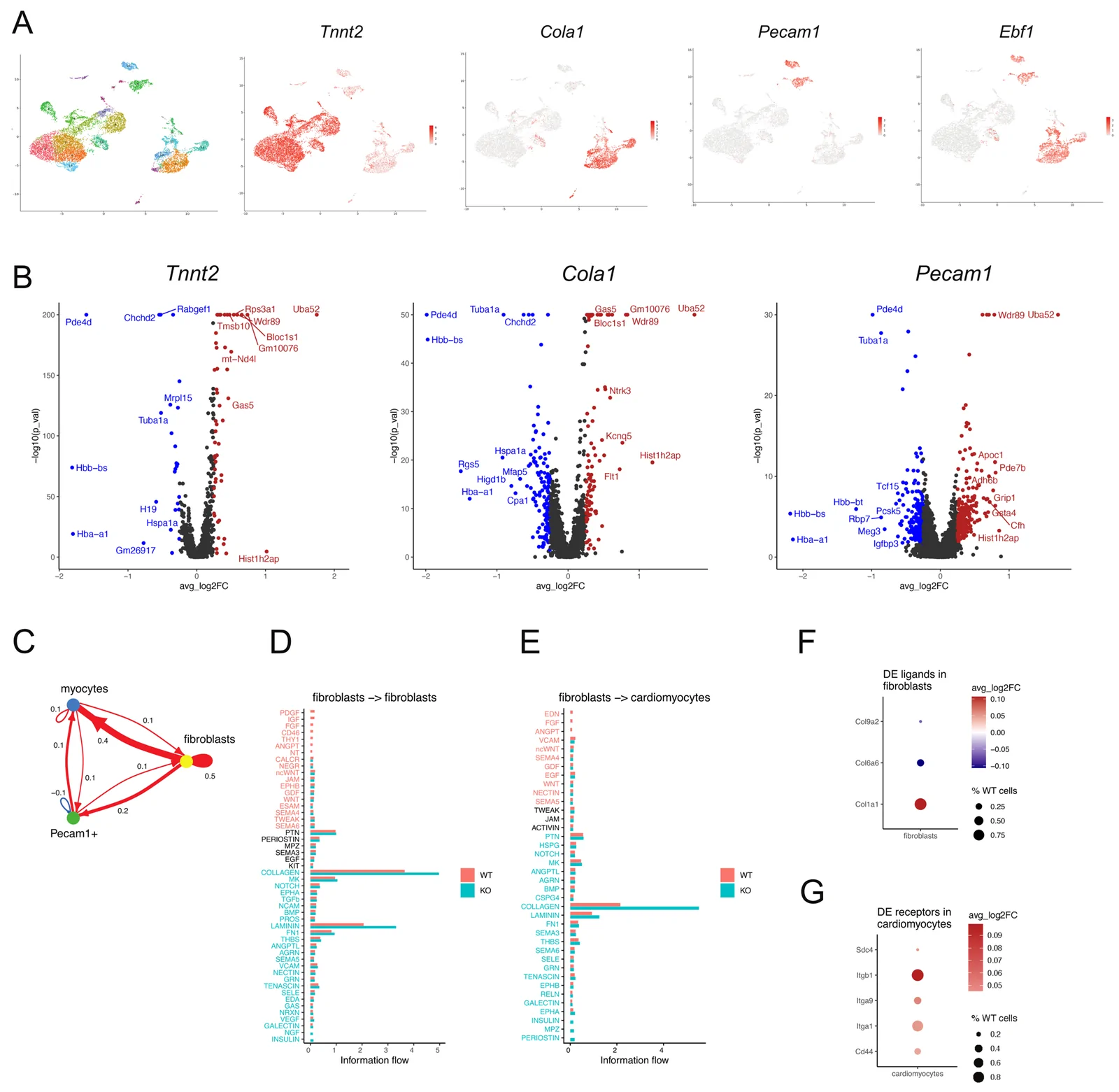
**Enhanced collagen signaling in EBF1 KO fibroblasts.** (**A**) UMAP plots of single-cell sequence-based clustering of E16.5 heart cells (n = 2 hearts; 12,235 cells) showing 22 distinct clusters. Gene plots showing heatmap representation of gene expression for *Tnnt2* (myocytes), *Col1a1* (fibroblasts), *Pecam1* (endocardial/endothelial cells), and *Ebf1*. (**B**) Volcano plots for EBF1 KO DEGs from *Tnnt2*^+^, *Col1a1*^+^, and *Pecam1*^+^ cells. (**C**) CellChat analysis of differential interaction strength between myocytes, fibroblasts, and *Pecam1*^+^ cells. Red arrows denote enhanced information strength. Blue arrows denote diminished information strength. (**D**,**E**) Bar graphs representing components of intercellular signaling from fibroblasts to fibroblasts (**D**) and fibroblasts to myocytes (**E**). (**F**,**G**) Heatmap of differentially expressed fibroblast ligands (**F**) and myocyte receptors (**G**) that comprise components of the collagen signaling pathway.

To deepen our understanding of the role of EBF1 in heart development, we first examined Ebf1 expression at single-cell resolution in dissociated cells isolated from embryonic day 16.5 hearts. In total, 22 distinct clusters of cells were identified (Figure 1A). Gene expression analysis identified three broad categories that accounted for the vast majority of cells: (1) *Tnnt2*-expressing cardiomyocytes, (2) *Col1a1*-expressing fibroblasts, and (3) *Pecam1*-expressing endocardial/endothelial cells (Figure 1A). Confirming prior observations [4], significant expression of EBF1 was seen only in fibroblasts and endocardial/endothelial cells (Figure 1A). Subsequently, differential gene expression analysis within these three broad categories of cells was performed comparing WT to KO cells. Amongst these three groups of cell types, KO fibroblasts demonstrated the largest changes in gene expression with 204 differentially expressed genes (DEGs), compared to 161 genes in *Pecam1*^+^ cells, and 77 genes in myocytes (Figure 1B). Interestingly, myocyte gene expression was significantly affected despite the lack of *Ebf1* gene expression in this cell type, consistent with a non-cell autonomous mechanism of action.

Based on prior observations implicating a paracrine mechanism for the effect of EBF1 on cardiac growth [4], we employed computational methods to predict all intercellular signaling pathways between the three main cell types in a systematic and unbiased fashion. CellChat [19] quantifies the signaling communication probability between two cell groups to analyze receptor–ligand interactions based on a simplified mass-action-based modeling. Using this methodology, significant changes in the interaction strength between the three main cell clusters were observed (Figure 1C). The largest difference in interaction strength was observed between fibroblasts and other fibroblasts, driven largely by increased collagen and laminin signaling (Figure 1D). Amongst different cell types, the fibroblast-to-myocyte intercellular interaction strength was the most affected and resulted in stronger information flow. This was driven largely by differences in the information flow related to the collagen signaling pathway (Figure 1E). Among the differentially expressed ligands expressed by fibroblasts, we found significantly higher *Col1a1* expression in KO cells (Figure 1F), consistent with an activated fibroblast phenotype. Interestingly, receptor expression analysis amongst myocytes revealed enhanced expression of many integrin receptor subunits (Figure 1G). In particular, the β1-integrin receptor (*Itgb1*) was significantly upregulated. Fibroblast-derived collagen signaling is a potent regulator of myocardial development [26], acting principally through the β1-integrin receptor. From this analysis, we conclude that EBF1 regulates fibroblast-to-myocyte signaling through the collagen–integrin pathway.

**Figure 2:**
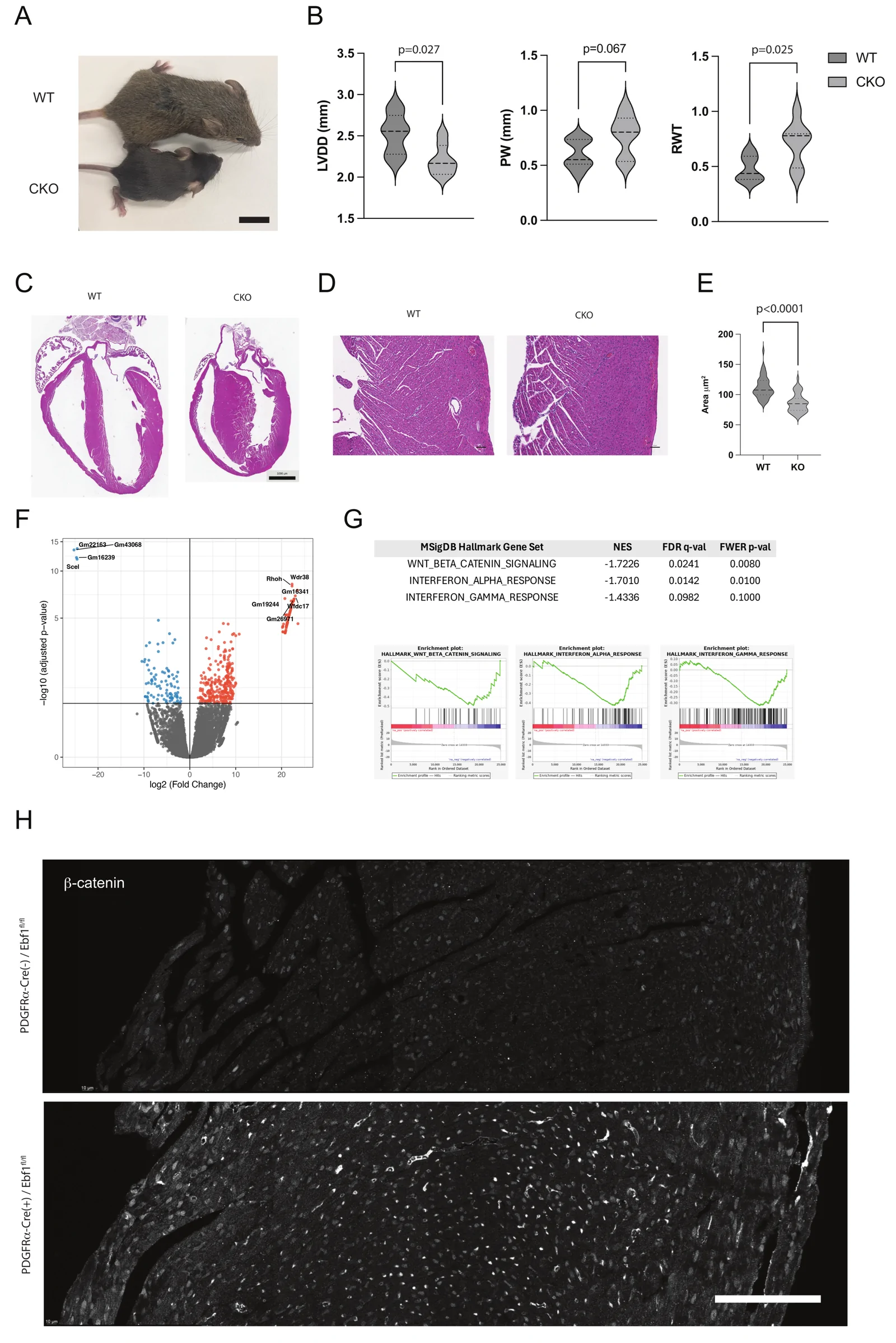
**Conditional knockout of EBF1 in fibroblasts.** (**A**) Photograph of WT (PDGFRα-Cre^−^/Ebf1^fl/fl^) and CKO mice (PDGFRα-Cre^+^/Ebf1^fl/fl^) at postnatal day 16. Scale bar is 500 mm. (**B**) Echocardiographic measurements of left ventricular diastolic diameter (LVDD), posterior wall thickness (PW), and relative wall thickness (RWT) in WT (n = 7) and CKO (n = 7) mice. (**C**,**D**) Representative H&E staining of WT and CKO heart sections at (**C**) low magnification and (**D**) high magnification. (**E**) Violin plot of myocyte cell size in WT (n = 2 hearts; 50 myocytes each) and CKO (n = 2 hearts; 50 myocytes each). (**F**) Volcano plot of DEGs from PDGFRα-GFP^+^ cells isolated from WT (n = 3) and CKO (n = 3) hearts. Significantly downregulated transcripts are shown in blue; significantly upregulated transcripts are shown in red. (**G**) Table of significantly enriched gene sets identified after GSEA analysis of DEGs and histograms of enriched gene sets from GSEA analysis. (**H**) Immunofluorescent staining of β-catenin in transmural sections of the interventricular septum of WT and CKO mice, showing increased nuclear accumulation in mutant hearts. Scale bar is 100 μm.

To experimentally validate our computational predictions implicating EBF1 expression in cardiac fibroblasts as a potent regulator of cardiac growth and development, we generated a fibroblast-specific conditional knockout mouse line using a PDGFRα-Cre driver line crossed with mice harboring a floxed EBF1 allele. Homozygous EBF1 conditional knockout (CKO) mice displayed an overt phenotype comparable to the generalized knockouts, including runting (Figure 2A). Additionally, similar to the generalized knockout, all homozygous CKO animals died prior to postnatal day 21.

Initial phenotypic assessment by echocardiographic assessment of animals at ~2 weeks of age revealed that the majority of EBF1 CKO hearts displayed smaller internal left ventricular diameters, but preserved left ventricular wall thicknesses, resulting in a significant increase in relative wall thickness, similar to the generalized knockout (Figure 2B). This behavior was bimodal, with some animals demonstrating normal heart morphology, while others were markedly thickened, indicative of incomplete penetrance of the phenotype. Similarly, histological examination of EBF1 CKO hearts revealed this same abnormal morphology, with a significant increase in relative wall thickness in some animals (Figure 2C,D), while others were relatively preserved. Hearts with significantly increased wall thickness were assessed for cell size to ascertain whether the basis for relative cardiac enlargement was increased cell size or cell number. We found that cardiomyocytes from EBF1 CKO hearts were significantly smaller than control hearts (Figure 2E), suggesting hyperplasia as opposed to hypertrophy as the underlying mechanism. These findings in the CKO mice of increased relative wall thickness, smaller cell size, runting, and premature death are similar to the generalized KO. Taken together, these data establish that fibroblast-specific loss of EBF1 is sufficient to potently regulate myocardial growth and development.

To further assess the impact of fibroblast-specific knockout of EBF1 on cardiac fibroblast gene expression, we introduced a PDGFRα-GFP allele into these mice, allowing us to label and isolate fibroblasts from dissociated heart preparations. FACS sorted GFP+ cells isolated from adolescent WT and EBF1 CKO mice were then subjected to bulk transcriptional profiling. In total, there were 450 DEGs, with 97 upregulated and 353 downregulated genes in the EBF1 CKO fibroblasts. (Figure 2F). GSEA revealed significant enrichment of components of the Wnt signaling pathway as well as interferon signaling in EBF1 CKO fibroblasts (Figure 2G). To experimentally test whether dysregulated Wnt signaling was observed in the EBF1 CKO hearts, β-catenin staining was performed. Significantly more intense nuclear staining was seen in a subset of mutant hearts compared to WT littermate controls (Figure 2H). Wnt signaling in fibroblasts is a potent regulator of activation, proliferation, migration, and ECM production [27], and its excessive nuclear expression suggests an activated fibroblast phenotype. Persistent activation of this developmental signaling pathway into adolescence suggests a failure of proper fibroblast maturation.

Intercellular communication between fibroblasts and cardiomyocytes has been well established in both normal development and pathological states, acting through both paracrine mechanisms as well as via regulation of the extracellular matrix. Paracrine mediators include TGF-β, FGF2, and members of the interleukin family of cytokines, while ECM components such as collagen and fibronectin have been shown to regulate cardiomyocyte behavior through β1-integrin signaling [26,28]. Taken together, our findings support a model in which EBF1 normally serves to negatively regulate fibroblast activation and the magnitude of collagen-dependent myocardial signaling. In the absence of EBF1, our data further suggest that fibroblast-derived collagen-to-cardiomyocyte β1-integrin receptor (*Itgb1*) signaling is unrestrained, leading to excessive cardiomyocyte proliferation and defects in cardiac growth and development. These data lend further support to the concept that EBF1 regulates the maturation of numerous complex multicellular tissues through heterocellular signaling cascades. Whether this signaling cascade is dysregulated in response to various cardiac stressors, such as hemodynamic overload, ischemia, or other pathologies, remains to be explored.

## Data Availability

The original contributions presented in this study are included in the article. Further inquiries can be directed to the corresponding author.

## Abbreviations

The following abbreviations are used in this manuscript:

EBF1	Early B-cell factor 1
PDGFRα	Platelet-derived growth factor receptor alpha
CKO	Conditional knockout

## References

[1] Lin H., Grosschedl R. (1995). Failure of B-cell differentiation in mice lacking the transcription factor EBF. Nature.

[2] Fretz J.A., Nelson T., Velazquez H., Xi Y., Moeckel G.W., Horowitz M.C. (2014). Early B-cell factor 1 is an essential transcription factor for postnatal glomerular maturation. Kidney Int..

[3] Zee T., Boller S., Györy I., Makinistoglu M.P., Tuckermann J.P., Grosschedl R., Karsenty G. (2013). The transcription factor early B-cell factor 1 regulates bone formation in an osteoblast-nonautonomous manner. FEBS Lett..

[4] Kim E.E., Shekhar A., Ramachandran J., Khodadadi-Jamayran A., Liu F.Y., Zhang J., Fishman G.I. (2023). The transcription factor EBF1 non-cell-autonomously regulates cardiac growth and differentiation. Development.

[5] Razy-Krajka F., Lam K., Wang W., Stolfi A., Joly M., Bonneau R., Christiaen L. (2014). Collier/OLF/EBF-dependent transcriptional dynamics control pharyngeal muscle specification from primed cardiopharyngeal progenitors. Dev. Cell.

[6] Treiber T., Mandel E.M., Pott S., Gyory I., Firner S., Liu E.T., Grosschedl R. (2010). Early B cell factor 1 regulates B cell gene networks by activation, repression, and transcription- independent poising of chromatin. Immunity.

[7] Boller S., Ramamoorthy S., Akbas D., Nechanitzky R., Burger L., Murr R., Schubeler D., Grosschedl R. (2016). Pioneering Activity of the C-Terminal Domain of EBF1 Shapes the Chromatin Landscape for B Cell Programming. Immunity.

[8] Wang Y., Zolotarev N., Yang C.Y., Rambold A., Mittler G., Grosschedl R. (2020). A Prion-like Domain in Transcription Factor EBF1 Promotes Phase Separation and Enables B Cell Programming of Progenitor Chromatin. Immunity.

[9] Liu X., Dai K., Zhang X., Huang G., Lynn H., Rabata A., Liang J., Noble P.W., Jiang D. (2023). Multiple Fibroblast Subtypes Contribute to Matrix Deposition in Pulmonary Fibrosis. Am. J. Respir. Cell Mol. Biol..

[10] Armartmuntree N., Murata M., Techasen A., Yongvanit P., Loilome W., Namwat N., Pairojkul C., Sakonsinsiri C., Pinlaor S., Thanan R. (2018). Prolonged oxidative stress down-regulates Early B cell factor 1 with inhibition of its tumor suppressive function against cholangiocarcinoma genesis. Redox Biol..

[11] Xing M., Ooi W.F., Tan J., Qamra A., Lee P.H., Li Z., Xu C., Padmanabhan N., Lim J.Q., Guo Y.A. (2020). Genomic and epigenomic EBF1 alterations modulate TERT expression in gastric cancer. J. Clin. Investig..

[12] Jimenez M.A., Akerblad P., Sigvardsson M., Rosen E.D. (2007). Critical role for Ebf1 and Ebf2 in the adipogenic transcriptional cascade. Mol. Cell. Biol..

[13] Pagani F., Tratta E., Dell’Era P., Cominelli M., Poliani P.L. (2021). EBF1 is expressed in pericytes and contributes to pericyte cell commitment. Histochem. Cell Biol..

[14] Song H., Zhang L.L., Zhong W.Q., Chen E.Q., Qiu X.X., Tang Z.L., Liao X.H. (2025). EBF1 expressed in the dermal papilla regulates hair type and length. Genes. Dis..

[15] Vilagos B., Hoffmann M., Souabni A., Sun Q., Werner B., Medvedovic J., Bilic I., Minnich M., Axelsson E., Jaritz M. (2012). Essential role of EBF1 in the generation and function of distinct mature B cell types. J. Exp. Med..

[16] Hamilton T.G., Klinghoffer R.A., Corrin P.D., Soriano P. (2003). Evolutionary divergence of platelet-derived growth factor alpha receptor signaling mechanisms. Mol. Cell. Biol..

[17] Choudhary S., Satija R. (2022). Comparison and evaluation of statistical error models for scRNA-seq. Genome Biol..

[18] Stuart T., Butler A., Hoffman P., Hafemeister C., Papalexi E., Mauck W.M., Hao Y., Stoeckius M., Smibert P., Satija R. (2019). Comprehensive Integration of Single-Cell Data. Cell.

[19] Jin S., Plikus M.V., Nie Q. (2025). CellChat for systematic analysis of cell-cell communication from single-cell transcriptomics. Nat. Protoc..

[20] Pallante B.A., Giovannone S., Fang-Yu L., Zhang J., Liu N., Kang G., Dun W., Boyden P.A., Fishman G.I. (2010). Contactin-2 expression in the cardiac Purkinje fiber network. Circ. Arrhythm. Electrophysiol..

[21] Kim E.E., Shekhar A., Lu J., Lin X., Liu F.Y., Zhang J., Delmar M., Fishman G.I. (2014). PCP4 regulates Purkinje cell excitability and cardiac rhythmicity. J. Clin. Investig..

[22] Dobin A., Davis C.A., Schlesinger F., Drenkow J., Zaleski C., Jha S., Batut P., Chaisson M., Gingeras T.R. (2013). STAR: Ultrafast universal RNA-seq aligner. Bioinformatics.

[23] Anders S., Pyl P.T., Huber W. (2015). HTSeq--a Python framework to work with high-throughput sequencing data. Bioinformatics.

[24] Love M.I., Huber W., Anders S. (2014). Moderated estimation of fold change and dispersion for RNA-seq data with DESeq2. Genome Biol..

[25] Quinlan A.R., Hall I.M. (2010). BEDTools: A flexible suite of utilities for comparing genomic features. Bioinformatics.

[26] Ieda M., Tsuchihashi T., Ivey K.N., Ross R.S., Hong T.T., Shaw R.M., Srivastava D. (2009). Cardiac fibroblasts regulate myocardial proliferation through beta1 integrin signaling. Dev. Cell.

[27] Jin X., Wang J., Cao R., Jiang D. (2026). Wnt Signaling Pathway: Biological Function, Diseases, and Therapeutic Interventions. MedComm.

[28] Kakkar R., Lee R.T. (2010). Intramyocardial Fibroblast Myocyte Communication. Circ. Res..

